# Retail Price and Point of Sale Display of Tobacco in the UK: A Descriptive Study of Small Retailers

**DOI:** 10.1371/journal.pone.0029871

**Published:** 2012-01-05

**Authors:** Dionysis Spanopoulos, Elena Ratschen, Ann McNeill, John Britton

**Affiliations:** 1 UK Centre for Tobacco Control Studies, University of Nottingham, Nottingham, United Kingdom; 2 Division of Epidemiology and Public Health, University of Nottingham, Nottingham, United Kingdom; Centre for Addiction and Mental Health, Canada

## Abstract

**Background:**

Since the implementation of the 2002 Tobacco Advertising and Promotion Act, point-of-sale (PoS) tobacco displays are one of few remaining means of communication between the tobacco industry and customers in the UK. This study aimed to explore the characteristics of tobacco displays in a UK city, and particularly to assess the tobacco prices and promotional offers, types and pack sizes on display.

**Methods:**

Digital pictures of PoS displays were taken in 117 small retail shops in Nottingham in mid 2010. Data were analysed using Windows Photo Gallery software and SPSS version 16.

**Results:**

Just over half (52%) of cigarette packs on display were packs of 20, and 43% packs of 10. Cigarette prices differed substantially between brands, ranging from £4.19 to £6.85 for 20-packs, and from £2.12 to £3.59 for 10-packs. Forty four percent of cigarette packs and 40% of RYO (Roll-Your-Own) tobacco pouches, almost exclusively lower priced brands, were displayed with a pricemark, implying a promotional price offer. Eighty percent of 20-pack cigarette brand or brand variants on sale were priced below the EU-defined Most Popular Price Category (MPPC) for the UK in 2010; 45**%** were priced below the Weighted Average Price (WAP), which replaced the MPPC in 2011.

**Conclusion:**

PoS displays communicate value by displaying a high proportion of lower cost brands, and smaller and hence lower-cost packs, and by displaying price discounts on packs. The MPPC substantially overestimated the prices at which most 20-cigarette packs were available. Removal of PoS displays will prevent this means of price marketing but our study also suggests that minimum pricing of 20-pack cigarettes, prohibition of sale of cigarettes in packs less than 20, and plain packaging to prevent pricemarking are necessary if price is to be used effectively as a tobacco control measure.

## Introduction

Price and advertising are major determinants of tobacco use. In the United Kingdom (UK), cigarette prices are regarded to be among the highest in Europe [1] and since the implementation of the Tobacco Advertising and Promotion Act (TAPA) in 2002 [2] tobacco advertising is now widely prohibited. Displays of cigarettes and other tobacco products at the PoS were exempt from this advertising legislation, although under the Health Act [3] are now to be prohibited in a phased manner from April 2012 to April 2015. Previous studies have shown that PoS tobacco displays are carefully managed by the tobacco industry [4], and that exposure stimulates unplanned purchases of cigarettes by smokers [5], [6], undermines quitting [5], [7], and is associated with adolescent smoking [8]. To date however, little is known about the characteristics of tobacco displays in retail outlets, or the product prices they offer to consumers.

We have therefore studied the characteristics of PoS tobacco displays, and the prices of cigarettes on sale, in a sample of local retailers in a UK city. In particular, we aimed to describe the types of tobacco products, the range of pack sizes, and the nature and extent of price promotions on display; to describe the mean and range of retail prices charged for tobacco products in practice; to explore the impact of price discounting on actual prices paid; and to describe the proximity of tobacco at PoS to products likely to be attractive to children, as well as the visibility of tobacco displays from outside the store.

## Methods

### Ethics

Ethics approval for the study was obtained from the University of Nottingham Medical School Ethics Committee.

### Data Collection

We identified all small tobacco retailers in residential areas of the combined Nottingham and West Bridgford conurbation listed in online local directories (192.com and yell.com), by searching for “convenience stores”, “newagents” and “off-licences”, which are the most common type of small stores selling tobacco products in the UK. We then attempted to visit all of them to determine whether tobacco products were sold there, and if so, to identify the owner or senior staff member on site. To this person we provided a verbal explanation of the study and requested signed consent to take photographs and collect other data on the retail tobacco display. An inconvenience fee of £20 was offered to all shopkeepers who provided signed consent. We also included in the study the small number of eligible retailers that were not identified in our directory searches but were observed in the process of the study. For stores that were part of larger chains or franchises, if requested by the manager, we made telephone and written requests to the relevant head office to seek authorization to participate.

With consent we took digital photographs of the PoS display to allow us to identify the brand, pack size and price of all products on display. An observational check list was used to assess the distance between tobacco displays and sweets or other products likely to be attractive to children, defining placement of children's products on or next to the sales counter in front of the tobacco display, or next to the tobacco display on the wall behind the sales counter, as ‘close proximity’. We also determined whether these products were located in the same visual field as tobacco when standing in front of them, and whether tobacco products on display could be seen from outside the store. Data were collected between June and September 2010.

### Data analysis

Photographs from each shop were examined using Windows Live Photo Gallery software to identify and record the total number of tobacco packs visible on display, and identify the type of tobacco (cigarette, cigar, Roll-Your-Own (RYO), pipe tobacco, other), and the brand name and pack size. We used these data to estimate the proportion of different categories of products (such as 20 and 10 packs of cigarettes, 25 and 12.5 gram RYO pouches) on each display. Where possible, we also determined the price of each pack on display, and what proportion of packs carried a price promotion (known as a pricemark) printed on its wrapper, or any other form of price promotion.

For the pricing analysis, we included one price for each brand, or where multiple variants of the same brand (eg *JPS Menthol, JPS Blue, JPS Silver*) were displayed, one price for each brand variant, irrespective of the number of packs of each brand or brand variant on display. When otherwise identical packs of the same brand or brand variant were displayed at different prices on the same display (usually because some packs were pricemarked and others not) we took the lowest price for analysis. We used these data to estimate the mean and standard deviation of tobacco prices across the retailers involved in the study. For descriptive purposes we grouped cigarette sale prices into 20-pence intervals. We also determined which cigarette brands were more commonly pricemarked, and the relation between the occurrence of pricemarking and the average undiscounted sale price of each brand; for simplicity in this analysis we grouped together variants of the same brand that typically sold at the same price. Descriptive statistics were produced using SPSS 16.0. The Most Popular Price Category (MPPC) price for March 2010 (and hence applicable during the period of data collection) was obtained from Tobacco Manufacturers' Association website [9].

## Results

**Figure 1:**
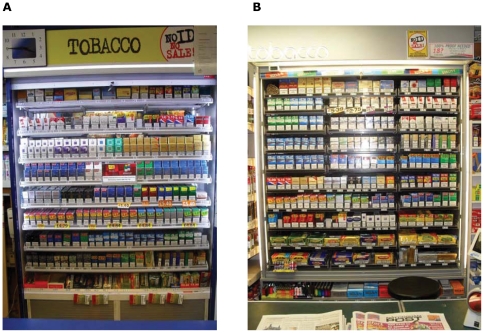
Examples of typical tobacco displays analysed in the study.

We were able to locate and visit 208 retailers in our study area, and obtained consent to collect data from 117 (56%). Since none of the head offices of the chain or franchise stores in the sample provided confirmation of consent to participate, all but two of the shops studied were independent small retailers. We took 1020 digital pictures of displays in these 117 study shops, from which a total of 14,031 displayed tobacco products were identified. These comprised 11,791 packs of cigarettes (including 96 multipacks typically containing 100 or 40 cigarettes), 1,431 of RYO tobacco, 666 packs of cigars (including multipacks and cigars for single sale), 61 of pipe tobacco, and 82 that were not clearly identifiable. On average, there were 120 (SD 50) tobacco products on display in shops, with a range from 27 to 250. The predominant form of price discounting we observed was pricemarking of individual packs, which was sometimes also highlighted by price tags placed on display shelves. In view of the relatively small numbers of pipe and cigar products on display, further analysis is restricted to packs of cigarettes and RYO tobacco. Examples of typical displays are shown in Figure 1.

### Cigarettes

Of the 11,695 single cigarette packs on display just over half (6052, 52%) were packs of 20, and 5050 (43%) were packs of 10. There were also 392 (3%) 19 packs, and 201 (2%) packs containing 24, 18, 16 or 14 cigarettes. On individual displays, a mean 45% (SD 10%) of all cigarette single packs on display were packs of ten, with a range from 27% to 90%.

**Figure 2:**
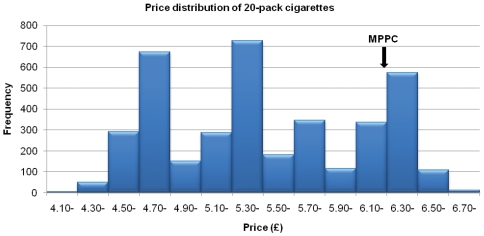
Price distribution of 20-pack cigarettes.

We analysed sale prices for 20 and 10-pack cigarettes. Of the 6052 packs of 20 cigarettes on display, 2037 represented duplicates or multiples of the same brand or the same brand variant on the same display. Of the 4015 brands or brand variants that appeared at least once on different retail displays we were able to identify a price for 3861 (96%). The mean (SD) price of these 20-pack cigarettes was £5.50 (£0.62) and the range from £4.19 to £6.85; however, prices were clustered around three price modes (low, mid and high) of £4.70–£4.89, £5.30–£5.49 and £6.30–£6.49 (Figure 2). The MPPC price for 20 cigarettes in March 2010 was £6.29 [9], which was higher than 80% of the displayed brand or brand variant prices we observed, and higher than the mid- and low-price modes by around £0.80 and £1.60. Of the 5050 packs of ten cigarettes on display, 2008 were duplicates or multiples on the same display, leaving 3042 brands or brand variants that appeared once or more on different displays. We were able to identify a price for 2905 (95%) of these; the mean (SD) was of £2.86 (£0.33), and the range from £2.12 to £3.59.

**Figure 3:**
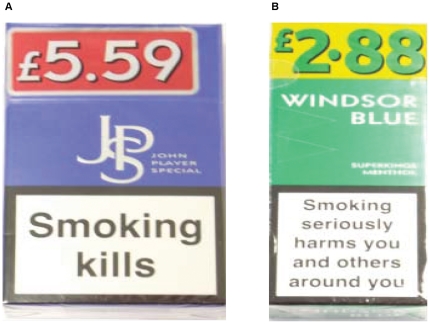
Examples of price-marked cigarette packs.

Nearly half (44% of 20-packs and 42% of 10-packs) of all cigarette packs on display were pricemarked (see figure 3 for examples), as were 86% of the only brand sold in 19-packs (*Pall Mall*). Pricemarking was observed in almost all of the shops we visited, and was limited to lower price brands; no brand that retailed at over £5.70 for 20 (or £2.95 for 10) was pricemarked. The proportion of pricemarked packs for the most commonly displayed brands in relation to undiscounted price is shown in Table 1; the proportion increases from 0% for the eight most expensive brands, to at least 70% for the nine least expensive, and the cheapest brand (*Park Road*) being available only in pricemarked packs. Relative to undiscounted packs of the same brand pricemarking typically offered a discount of between 2 and 11% (Table 1).

**Table 1:** Price ranges, average undiscounted and pricemarked price, discount in pence and as a percent of undiscounted price, number of packs on display and the proportion of pricemarked packs for the most commonly displayed 20-pack brands or similarly priced brand variants, ranked in descending undiscounted price order (NA = not available or not applicable).

BRANDS	PriceRange	AverageRegularPrice	AveragepricemarkedPrice	Discount(pence)	Discount(%)	Number ofpacksdisplayed	Pricemarked(%)
**Embassy**	6.1–6.77	6.37	NA	NA	NA	225	0
**Marlboro**	5.85–6.87	6.37	NA	NA	NA	293	0
**Silk Cut**	6.15–6.59	6.36	NA	NA	NA	414	0
**Benson & Hedges Gold**	5.98–6.6	6.35	NA	NA	NA	293	0
**Camel**	6.15–6.5	6.33	NA	NA	NA	58	0
**Lucky Strike**	5.68–6.85	6.32	NA	NA	NA	23	0
**Regal**	5.99–6.5	6.31	NA	NA	NA	27	0
**Superkings**	5.99–6.57	6.18	NA	NA	NA	231	0
**Berkeley**	5.35–6.57	6.02	5.4	62	10	157	33
**Benson & Hedges Silver**	5.55–6.35	5.84	5.68	16	3	230	18
**L&B**	5.6–6.39	5.82	NA	NA	NA	445	0
**Marlboro Bright Leaf**	5.39–6.47	5.81	5.58	23	4	91	88
**JPS Black(White)**	4.42–6.03	5.6	4.96	64	11	89	2
**Sovereign**	5.2–5.88	5.59	NA	NA	NA	131	0
**Richmond**	4.99–5.87	5.47	5.33	14	3	753	76
**Mayfair**	4.8–5.79	5.43	5.25	18	3	885	70
**Royals**	4.67–6.1	5.31	5.05	26	5	196	79
**Winston**	4.79–5.19	5.19	4.79	40	8	29	93
**Sterling**	4.37–5.19	4.88	4.77	11	2	615	79
**JPS Silver(Menthol,Blue)**	4.3–5.9	4.87	4.75	12	2	326	89
**Windsor Blue**	4.35–5.33	4.87	4.74	13	3	274	82
**Red Band**	4.25–4.8	4.76	4.6	16	3	111	95
**Park Road**	4.33–4.49	NA	4.48	NA	NA	24	100

Pricemarking of 10-pack cigarettes showed a similar association with price, and offered proportionately similar discounts to those on 20-pack cigarettes; results are not therefore presented in detail. Pricemarked 10-pack cigarettes were typically the lowest priced cigarettes, and represented 18% of the total number of cigarette packs on display.

Multipacks were observed in only 7 shops, and offered discounts of up 1 to 22 pence for 20 cigarettes, or under 5% of the undiscounted price of 20-packs of the same brand or brand variant in the same shop.

### RYO Tobacco

Of 1431 RYO tobacco pouches identified on display, the majority (862, 60%) were packs of 12.5 g, and 429 (30%) of 25 g. The remainder comprised 91 (6%) 50 g, 21 (1%) 14 g, and 28 (2%) 10 g pouches. The two least expensive RYO tobacco pouch sizes (10 and 12.5 gr) accounted for 62% of the total RYO tobacco pouches displayed in all shops, with a range from 25 to 100% in individual shops. We analysed price for the two most common RYO tobacco categories, 12.5 g and 25 g. We were able to identify prices for 704 (93%) 12.5 g RYO brand or brand variants appearing at least once on different displays with a mean (SD) of £3.06 (£0.20) and range from £2.00 and £3.65. For the equivalent 378 (91%) 25 g RYO brands, a mean (SD) price was £5.96 (£0.41) and the range from £3.69 to £7.22.

A total of 571 (40%) RYO tobacco pouches were pricemarked, including 41% of 50 g pouches, 34% of 25 g pouches, 42% of 12.5 g pouches, and 86% of 10 g pouches. No 25 g RYO brand retailing at over £5.80, or over £3.25 for 12.5 g, was pricemarked. Pricemarking typically offered a discount of between 2 and 6% on the regular price.

### Visibility of tobacco products from outside the shop, and proximity to products aimed at children, and prominence of low cost brands on display

**Figure 4:**
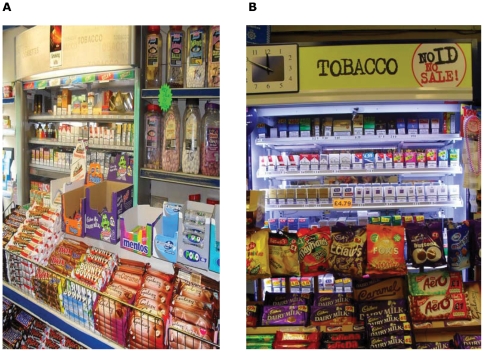
Tobacco displays in close proximity and in the same visual field with sweets.

In 65% of stores, at least some of the tobacco display was visible from outside the shop. Products attractive to children (chocolates and sweets) were placed at close proximity to tobacco displays in 85 (73%) shops, and were judged to be in the same field of vision in 76 (65%; figure 4). We did not formally analyse the positioning of brands by price or pricemarking, though visual inspection of the display images indentified no consistent pattern in the placing of higher price (and hence undiscounted) brands and lower priced brands. The proportion of price-marked packs of cigarettes and RYO tobacco on each display was unrelated to the local area deprivation, measured by the Index of Multiple Deprivation score [10], for the postcode of the store.

## Discussion

This study is the first to describe the price structure of tobacco products displayed at the PoS in the UK. We observed that cigarettes are available at a wide range of prices, and that 80% of the 20-pack prices were lower than the EU-defined Most Popular Price Category (MPPC). Modal mid- and low-price 20-pack cigarettes undercut the MPPC by around £0.80 and £1.60. Prices were even lower for 10-pack cigarettes, which typically accounted for nearly half of all cigarette packs on display; low cost packs of 12.5 g tobacco were also the predominantly displayed RYO tobacco. Cigarettes in the low and middle range of price were also frequently pricemarked, with prominent price labelling printed on the pack to promote what was typically a modest discount on the regular price. Our findings thus demonstrate that price is a major component of PoS promotion, and that the MPPC, which was the EU-defined measure of cigarette price applying at the time our data were collected, is unrepresentative of available cigarette prices. The MPPC has since been replaced by the Weighted Average Price [11], which for the UK in January 2011 was €6.27 (approx £5.37, or 90 pence less than the MPPC) and therefore much closer to the median (at approximately the 46^th^ percentile) of the range of prices we observed. However, 20-pack cigarettes were still available at the time of our study, in mid 2010, at over £1 less than the 2011 WAP.

Although this research was carried out in a single UK city, the ubiquity of PoS gantries internationally and the fact that they are often supplied or serviced by multinational tobacco companies indicates that our findings are likely to be generalisable across the UK and indeed to other EU countries with similar pricing and advertising legislation. Our study was restricted almost entirely to small retailers, as we were unable to obtain consent from the great majority of managers of bigger shops that were part of a local or national chains. However, since the larger volume of cigarettes sales takes part in small shops [12], and since the majority of underage retail purchasing of cigarettes occurs in small retailers [13], our findings are likely to be representative of the prices paid for cigarettes by price conscious groups including children. The widespread use of pricemarking to further promote the price advantage of middle- and lower-cost brands was consistent with data from other jurisdictions showing that price-based marketing is the leading means of promotional activity for cigarettes [14]. With the exception of a small number of multipacks, most of which also offered a modest discount but at a higher total purchase price, we did not observe any other forms of price promotion, though most of the options used widely in other countries, such as voucher schemes, are now illegal in the UK. Although the use of pricemarking of tobacco products has been discussed previously [15], [16], this study is the first to document the full extent to which pricemarking features in PoS displays. This means of promotion will persist for several more years in the UK as tobacco displays in small retailers will not be prohibited until 2015.

Price is a key component of tobacco control policy, and according to the UK government, the UK has some of the highest prices in Europe [1]. Our findings suggest that whilst this may be true of the MPPC (and now the WAP) in relation to other EU Member States, these measures do not reflect the prices at which cigarettes are available to smokers. For the most price conscious consumers, which arguably include children and young people [1], the widespread availability of 10 pack cigarettes and of 10 g or 12.5 g RYO pouches, retailing at little over £2.00, represent substantially lower price options that undermine the use of price as a tobacco control measure. The UK is one of the few EU countries in which the sale of 10-pack cigarettes is permitted, and 10-packs are more popular in the UK than in the few other EU countries in which they are still available [12], [17]. Ten pack cigarettes are also popular among pupils aged 11–15 who managed to buy cigarettes from a shop [13]. Retailers reported to us, as elsewhere (Convenience Store. Retailer's view, 15^th^ October 2010) that pricemarked products tended to sell more easily, suggesting that PoS displays are an effective advertising means of promoting discounted and thus cheaper tobacco products, even though the actual discount is modest. Nevertheless, a decrease in the relative prices of the lower priced-brands leads to an increase of their market share [18]. Economy brands account more than 64% of total cigarette sales in the UK, and this sector is expected to grow further in the next years [12]. These and our observations indicate that current UK price and packaging structures need to be reformed if the affordability of cigarettes is to be reduced.

Our findings also highlight the potential dangers of tobacco displays as an advertising medium, particularly as previous studies have shown that promotions and price influence smoking uptake among young people [19], that perceived low cost of cigarettes was related with regular smoking among teenagers [20] and the effectiveness of price on smoking cessation is undermined by the availability of cheaper cigarettes [21]. Our observation that tobacco products are commonly displayed close to and often in the same field of vision as products attractive to children reflects a high likelihood that children will notice and absorb information about tobacco products when visiting small shops. The visibility of tobacco displays from outside the shop, which is also common, is likely to help to attract potential consumers into the store.

Overall therefore our study demonstrates that PoS displays are a persistent medium by which cigarette prices can be and are communicated to existing or potential customers, through the availability of low price packages, such as 10 pack cigarettes and 10 or 12.5 g RYO tobacco, and through pricemarking. We conclude that if price is to be used effectively as a tobacco control measure, then in addition to PoS display prohibition, it is essential to set a minimum price for 20 cigarette packs, and to prohibit the sale of cigarettes in packs of less than 20. Our findings on the widespread use of pricemarking as a means of price promotion at the PoS supports the call for plain packaging and adds further justification for the prohibition of display of cigarette packs at PoS being implemented or considered in several countries, as it will prevent overt promotion of price through pricemarking.
